# Mechanism of Antibacterial Activity of *Bacillus amyloliquefaciens* C-1 Lipopeptide toward Anaerobic *Clostridium difficile*

**DOI:** 10.1155/2020/3104613

**Published:** 2020-03-03

**Authors:** Jia Lv, Rong Da, Yue Cheng, Xiaohong Tuo, Jie Wei, Kaichong Jiang, Adediji Omolade Monisayo, Bei Han

**Affiliations:** ^1^School of Public Health, Health Science Center, Xi'an Jiaotong University, Xi'an, China; ^2^Department of Clinical Laboratory, The First Affiliated Hospital of Xi'an Jiaotong University, Xi'an, China

## Abstract

Probiotics may offer an attractive alternative for standard antibiotic therapy to treat *Clostridium difficile* infections (CDI). In this study, the antibacterial mechanism *in vitro* of newly isolated *B. amyloliquefaciens* C-1 against *C. difficile* was investigated. The lipopeptides surfactin, iturin, and fengycin produced by C-1 strongly inhibited *C. difficile* growth and viability. Systematic research of the bacteriostatic mechanism showed that the C-1 lipopeptides damage the integrity of the *C. difficile* cell wall and cell membrane. In addition, the lipopeptide binds to *C. difficile* genomic DNA, leading to cell death. Genome resequencing revealed many important antimicrobial compound-encoding clusters, including six nonribosomal peptides (surfactins (srfABCD), iturins (ituABCD), fengycins (fenABCDE), bacillibactin (bmyABC), teichuronic, and bacilysin) and three polyketides (bacillaene (baeEDLMNJRS), difficidin (difABCDEFGHIJ), and macrolactin (mlnABCDEFGHI)). In addition, there were other beneficial genes, such as phospholipase and seven siderophore biosynthesis gene clusters, which may contribute synergistically to the antibacterial activity of *B*. *amyloliquefaciens* C-1. We suggest that proper application of antimicrobial peptides may be effective in *C*. *difficile* control.

## 1. Introduction

*Clostridium difficile* is an anaerobic, gram-positive, spore-forming bacterium. Clinical signs of *C. difficile* infection (CDI) range from mild diarrhea to fulminant colitis [1]. The incidence and severity of CDI have increased significantly, especially by the recently emerged and highly virulent epidemic strain BI/NAP1/027 [2]. With increasing antibiotic resistance of *C. difficile*, there is an urgent need to develop new agents and efficient methods for the treatment and control of CDI [1, 3]. Distinct from the traditional antibiotics, many novel antimicrobial agents, such as ramoplanin, surotomycin, and cadazolid, are currently being investigated in clinical trials for the treatment of CDI. Surotomycin is an orally dosed lipopeptide antibiotic that acts by disrupting the cell membrane [4].

Bacteriocins are ribosomally synthesized antimicrobial peptides with high activity against other bacteria. Bacteriocins are secreted by some probiotic microorganisms, such as *Lactobacillus* species, S*accharomyces boulardii*, and bifidobacteria [5]. Thus, further evaluation should be given to the bacterial resources, antimicrobial mechanisms, and biosafety of probiotics in considering bacteriocins as an alternative or adjunctive therapeutic method for CDI.

Except the ribosomally synthesized antimicrobial peptides, *Bacillus* species could synthetize a mixture of lipopeptides by nonribosomal peptide synthetases, which mainly include surfactin, iturin, lichenysin, and fengycin families, with broad-spectrum biological activities [6–8]. With the described functional secondary metabolites, many *Bacillus* spp. strains have been developed as biofertilizers and biopesticides, and they are currently regarded as promising environmentally friendly means for plant protection and plant growth promotion, and as secondary metabolite factories [9]. However, for *B. amyloliquefaciens*, there are only a few reports that describe an antimicrobial activity against toxin-producing *C*. *difficile* [10].

We isolated *B. amyloliquefaciens* C-1 from ready-to-eat fruit samples. The bacterial strain is stored in the China Center for Type Culture Collection as a patent strain with the number of CCTCC M2010177. The supernatant of C-1 showed high antioxidant activity and inhibitory activity against foodborne pathogens (*Escherichia coli* O157:H7, *B*. *cereus*, *S*. *aureus*, etc.) and human pathogens (*C. difficile*, *Klebsiella pneumoniae*, *Enterococcus faecium*, etc.) [11, 12]; however, no effect against fungi was found.

Comparative genomic analysis showed evolutionary traits for *B. amyloliquefaciens* strain adaptation to host habitats [13]. The C-1 strain exhibited a biosurfactant activity phenotype against pathogens. The molecular bases/mechanisms of this pathogen-specific activity were unknown. In this study, we investigated the anti-*C*. *difficile* mechanisms of the secreted *B. amyloliquefaciens* C-1 extracellular lipopeptides. We systematically investigated the effects of C-1 lipopeptide on *C. difficile* cell growth, morphological structure, cell wall and membrane integrity, and genome. Then, we resequenced the entire C-1 genome and identified relevant gene clusters, locations, and potential regulatory sequences, including genes for bacteriocins, ribosomally synthesized antibacterial peptides, phospholipase, siderophores, and genes that provide resistance to toxic compounds.

## 2. Material and Methods

### 2.1. Bacterial Strains and Culture

*B*. *amyloliquefaciens* C-1, a patent strain (Chinese patent no. ZL201410260574.2), was isolated and stored in our lab. It was inoculated into fermentation medium (12.4 g/l tryptone, 20 g/l glucose, 5 g/l NaCl, 1.5 g/l K_2_HPO_4_·3H_2_O, 0.04 g/l MnSO_4_·H_2_O, 1.7 g/l FeSO_4_·7H_2_O, and 1.2 g/l MgCl_2_·6H_2_O, pH 7.2) and grown with shaking of 200 rpm in flasks for 72 h at 30°C. *Clostridium difficile* ATCC 9689, 700057, and BAA-1870 strains were obtained from the American Type Culture Collection and stored at -80°C. *C*. *difficile* strains were cultured in sterile Reinforced *Clostridium* Medium (RCM) and incubated overnight at 35°C in an anaerobic chamber (Coy Laboratory Products Inc., Ann Arbor, Michigan) with an atmosphere of 82% N_2_, 15% CO_2_, and 3% H_2_.

### 2.2. Isolation and Identification of *B. amyloliquefaciens* C-1 Extracellular Lipopeptide

Lipopeptide isolation was performed by acid precipitation according to Zhang et al. [14]. Briefly, the crude cell-free culture was adjusted to pH 2.0 with 6 M HCl and placed overnight at 4°C. After centrifugation, the precipitate was extracted twice with methanol. The solution was dried in a vacuum freeze dryer, and the dry residue was dissolved in 50 mM Tris-HCl (pH 7.5) and passed through a 0.22 *μ*m filter. The extracted lipopeptide sample was analyzed with a UV-VIS spectrophotometer (UV5, Mettler Toledo).

### 2.3. Thin-Layer Chromatography

The purified lipopeptide was examined by thin-layer chromatography (TLC) on a silica gel G plate [15]. TLC assay was performed on a silica gel G plate (10 × 20 cm, Silica gel 60, Germany). A chloroform-methanol mixture (10 : 1, *v*/*v*) was used as the mobile phase. A sample was spotted onto the TLC plate and hydrolyzed with 6 M HCl for 2 h in a sealed container. Once dried, the plate was developed in the mobile phase. After development, the plate was sprayed with 0.5% ninhydrin and placed in an oven at 110°C for 10 min to detect the peptides as red spots.

### 2.4. Semipreparative HPLC Analysis

The putative lipopeptides were identified by HPLC analysis. Briefly, crude extract spots were removed from the TLC plates and dissolved in 10% methanol; the supernatants were analyzed by semipreparative high-pressure liquid chromatography using an Agilent LC 1200 system. The chromatographic separation was performed with a C-18 Column (4.6 × 250 mm). The column outlet was coupled to an Agilent MSD Ion Trap XCT mass spectrometer equipped with an ESI ion source. The lipopeptide fragments were selectively desorbed with methanol gradients from 35% to 65% within 140 min. All elution programs used a flow rate of 0.5 ml/min at 214 nm and detection occurred using the negative ion mode at *m*/*z* ranging from 400 to 2000. The isolated fragments were collected for the following experiments.

### 2.5. Detection of Lipopeptide Synthesis-Related Genes

Lipopeptide biosynthesis genes (*sfr*, *ituD*, and *fenB*) were identified by PCR (*sfr*-F: 5′ ATGAAGATTTACGGAATTTA 3′, *sfr*-R: 5′ TTATAAAAGCTCTTCGTACG 3′; *ituD*-F: 5′ ATGAACAATCTTGCCTTTTTA 3′, *ituD*-R: 5′ TTATTTTAAAATCCGCAATT 3′; *fenB*-F: 5′ CTATAGTTTGTTGACGGCTC 3′, *fenB*-R: 5′ CAGCACTGGTTCTTGTCGCA 3′) [9]. PCR conditions consisted of an initial denaturation step at 94°C for 5 min followed by 30 cycles of denaturation at 94°C for 1 min, 54°C annealing for 45 sec (*sfr*, *ituD*) or 1 min (*fenB*), and 72°C extension for 1 min followed by a final extension step at 72°C for 7 min. The amplified PCR product was purified and sequenced by an automated sequencer (3730 DNA Analyzer). PCR product sequences were identified using GenBank nucleotide data and BLAST from the National Center for Biotechnology Information, Bethesda, MD, USA (http://www.ncbi.nlm.nih.gov/blast/).

### 2.6. The Inhibitory Activity of Lipopeptide against *C. difficile*

Antimicrobial activities of the lipopeptides were detected by disc diffusion assay. 0.5 McF (10^6^ CFU/ml) of *C*. *difficile* cells was inoculated onto the surface of blood agar plates. An Oxford cup (6 mm diameter), containing 100 *μ*l lipopeptide with concentrations of 5, 10, and 15 *μ*g/ml dissolved in 10% methanol, was placed on test *C. difficile*-seeded plates. A cup containing 100 *μ*l 10% methanol was used as negative control. Each *C*. *difficile* strain was plated in triplicate. The plates were incubated anaerobically overnight at 35°C, and antimicrobial activity was evaluated by measuring inhibition zones against the tested *C*. *difficile* cells. The minimal inhibitory concentrations (MIC) against *C*. *difficile* strains were determined by the broth microdilution method in 96-well microplates with a final concentration of 10^5^ CFU/ml, and the final concentration of the added lipopeptide ranged from 10 *μ*g/ml to 0.0095 *μ*g/ml; bacterial growth without lipopeptide was set as control [16]. The MIC was defined as the lowest lipopeptide concentration at which growth was completely inhibited after overnight anaerobic incubation of the plates at 35°C.

### 2.7. Growth of *C. difficile* Incubated with Lipopeptides

For time-kill analyses, 0.5 McF (10^6^ CFU/ml) of *C*. *difficile* cells (strains ATCC 9689, ATCC 700057, and ATCC BAA-1870) was prepared and inoculated into fresh RCM containing 0, 0.25, 0.5, and 0.75 MIC of lipopeptide separately, and incubated in the anaerobic chamber at 35°C. Cell viability was determined every 2 h for 24 h [17]. Each treatment was performed with three biological replicates.

### 2.8. Scanning Electron Microscope Analysis of *C. difficile* Cells Treated with Lipopeptides

An overnight culture of *C*. *difficile* was transferred into fresh RCM medium with 0.5 McF and 0.25 MIC of lipopeptide, and incubated anaerobically at 35°C for 1 h. The *C. difficile* cells were collected and washed three times with sterile PBS solution. The cells were fixed with 2% glutaraldehyde overnight at 4°C and then dehydrated by a graded series of ethanol (50%, 70%, 80%, 90%, 95%, and 100%) for 20 min at each step. After the critical point of drying and gold coating, the surface structure of treated *C*. *difficile* cells was observed with a scanning electron microscope (Hitachi S-2460N, Hitachi, Ltd., Tokyo, Japan) at an acceleration voltage of 20 kV [18].

### 2.9. Fluorescence Microscope Analysis of *C. difficile* Cells Treated with Lipopeptides

Propidium iodide (PI) penetrates only damaged cell membranes, whereupon it binds to double-stranded DNA and fluoresces red with 488 nm illumination. To clearly detect an effect on the plasma membrane, 0.5 MIC of the purified lipopeptide was incubated with *C*. *difficile* cells (10^7^ cells/ml) in RCM liquid medium. The mixture was incubated anaerobically at 35°C for 1 h. Then, 10 *μ*l of 100 *μ*g/ml PI solution was added to the cell suspension, and the mixture was incubated for 30 min in the dark. Finally, the treated *C*. *difficile* cells were observed with a Nikon TI-S fluorescence microscope with the filters set at an excitation wavelength of 488 nm and an emission wavelength of 538 nm. Cells treated with the same amount of sterile water were used as a negative control [19]. All experiments were repeated three times.

### 2.10. Determination of Extracellular Alkaline Phosphatase Activity of *C. difficile* Cells Treated with Lipopeptide

An overnight culture of *C*. *difficile* was subcultured into fresh RCM liquid medium. Lipopeptide was added separately into the *C*. *difficile* culture at final concentrations of 0.25 and 0.5 MIC. Bacterial supernatant (0.5 ml) was collected every 12 h for the measurement of extracellular alkaline phosphatase (AKPase) activity using an AKP assay kit (Nanjing Jiancheng Technology Co., Ltd., Nanjing, China) as described in [20]. The AKPase unit was defined as 1 mg of phenol produced by 100 ml of bacterial culture supernatant reacted with the substrate at 37°C for 15 minutes. Cells treated with the same amount of sterile water were used as negative control. Each test was performed in three biological replicates.

### 2.11. Lipopeptide Binding to *C. difficile* Genomic DNA

Gel retardation experiment assays were performed to identify the DNA binding activity of the lipopeptide as described in [21]. Briefly, 50 ng of *C*. *difficile* genomic DNA was mixed with 1 *μ*l of 1, 2, and 5 *μ*g/ml lipopeptide in 20 *μ*l of binding buffer (10 mM Tris-HCl, 1 mM EDTA buffer, pH 8.0). One *μ*l of sterilized water mixed with *C. difficile* genomic DNA was used as negative control. Mixtures were incubated at 35°C for 1 h. All samples were subjected to 1.0% agarose gel electrophoresis and stained with ethidium bromide.

### 2.12. Whole Genome Sequencing of *B. amyloliquefaciens* C-1

The genomic DNA of strain C-1 was isolated and purified by a kit (Applied Biosystems® 4413021) and sequenced on the Pacific Bioscience (PacBio) RS II system at Genefund, Shanghai, China. The genome was assembled with SMRT analysis v.2.3 and RS_HGAP_Assembly.3, and the genome assembly was improved by using the software Pilon. Identification of protein-coding open reading frames (ORFs) and annotation of ORFs were performed by using the NCBI Prokaryotic Genome Annotation Pipeline. Genes were functionally annotated by BLAST search in COG (Gene Ontology Consortium), Nr (NCBI RefSeq), and Pfam Databases [22, 23]. KEGG (Kyoto Encyclopedia of Genes and Genomes) database was used in the analysis of metabolic pathways of lipopeptide-producing *Bacillus* species. All amino acid sequences derived from the *Bacillus* genomes were submitted to the KEGG database, and the metabolic functions of these sequences were annotated by KASS. The KO (KEGG Orthology) term and corresponding KEGG pathway for each ORF were automatically generated. Secondary metabolite clusters present in the genome of the *B. amyloliquefaciens* collection have been evaluated using antiSMASH 5.0 [24].

The 16S rRNA gene sequences of *Bacillus* species were extracted from genome sequences and aligned using the CLUSTALX [25]. Phylogenetic trees were constructed using the neighbor-joining method implemented in the software package MEGA version 7.0.26 [26]. Evolutionary distances were calculated using Kimura's two-parameter model.

The C-1 genome sequence data were deposited into the Sequence Read Archive (SRA) of NCBI and can be accessed via accession number SRP127533.

### 2.13. Statistical Analysis

All experimental data are expressed as the average with standard deviation of at least three independent replicates. Statistical analyses were performed using the *t*-test and analysis of variance (ANOVA), JMP pro (SAS Institute Inc., NC, US), STAMP^10^, and SPSS V20.0 (IBM Inc., IL, US). Significant differences were considered at *P* < 0.05.

## 3. Results and Discussion

### 3.1. Production, Purification, and Identification of C-1 Lipopeptide

**Figure 1:**
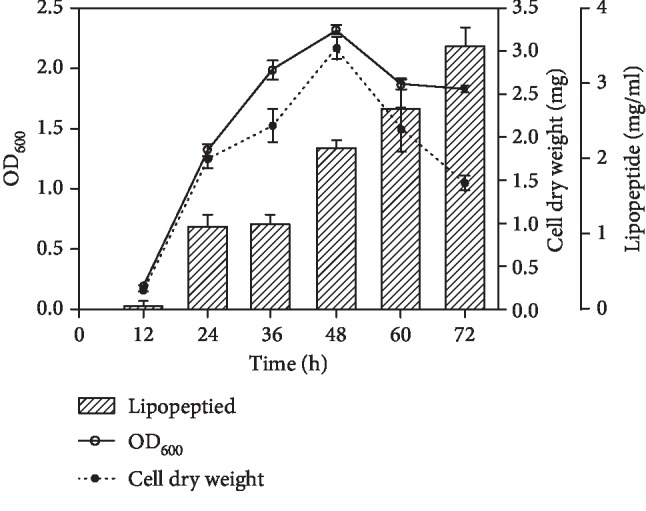
The fermentation and lipopeptide production in *B. amyloliquefaciens* C-1 for 72 hours. The solid line indicates the growth curve of OD_600_, the dotted line indicates the growth curve of cell dry weight, and the shaded columns represent the lipopeptide production.

*Bacillus* spp. produces abundant secondary metabolites, such as proteinase, amylase, bacteriocin, and exopolysaccharide. In this study, we focused on the lipopeptide-producing *B. amyloliquefaciens* strain C-1. The growth profile of C-1 is shown in Figure 1. The maximum growth and cell dry weight were reached at 48 h; the maximum lipopeptide production (3.49 ± 0.26 mg/ml) was reached at 72 h. Lipopeptide production was conducted in a fermentation medium. The active compound from the culture supernatant was scanned from 190 to 900 nm, and the maximum absorption occurred at 213 nm, which is the typical absorption wavelength of peptides. Three spots were observed by thin-layer chromatography, and three component peaks were detected by RP-HPLC. Mass spectroscopy showed that the molecular masses of the three components at *m*/*z* were 1067 Da, 1477 Da, and 1506 Da, which corresponded to surfactin, fengycin, and iurin, respectively. PCR products of 675 bp, 1400 bp, and 482 bp corresponded to *Srf*, *FenB*, and *ItuD* genes, and the PCR fragments were sequenced and showed 99% identity with surfactin, fengycin, and iturin biosynthesis gene clusters, individually ([Supplementary-material supplementary-material-1]).

### 3.2. Antimicrobial Activity of C-1 Lipopeptide against *C. difficile*

*Bacillus* spp. lipopeptides have an inhibitory activity against plant pathogenic fungi and pathogenic bacteria and have been developed as biocontrol agents [27]. Although, in our previous report, C-1 did not show any inhibitory activity against fungi, it did have antibacterial activity toward several human pathogens, and this antibacterial activity in the C-1 supernatant was verified to be the contribution of lipopeptide, not exopolysaccharide [11, 12].

**Figure 2:**
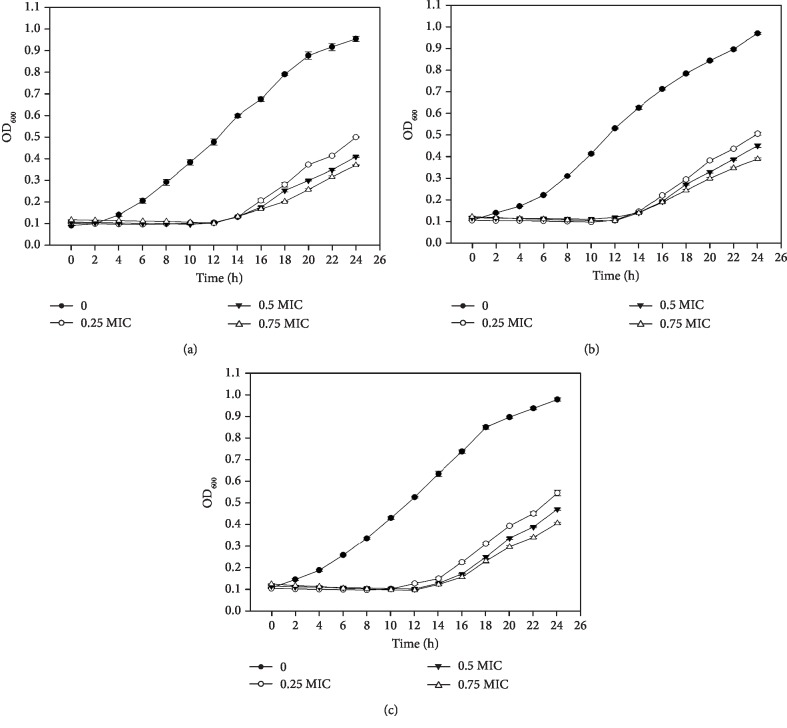
The growth curve of *C*. *difficile* ATCC 9689 (a), ATCC 700057 (b), and BAA-1870 (c) treated with different concentrations of the C-1 lipopeptide.

In plate tests, the C-1 lipopeptide displayed antagonistic activities against three *C*. *difficile* strains. Inhibition zone diameters ranged between 7.05 mm and 22.00 mm, and the largest inhibition zone was from 15 *μ*g/ml lipopeptide against strain *C*. *difficile* ATCC 9689 (Table 1). The MICs against *C*. *difficile* strains ATCC 9689, ATCC 700057, and ATCC BAA-1870 were 0.75, 2.5, and 2.5 *μ*g/ml, separately. Within a certain concentration range (0.0095 *μ*g/ml-10 *μ*g/ml), the inhibitory effect was positively correlated with the concentration of the C-1 lipopeptide. To analyze the inhibitory effect, we determined the growth curves of the three *C*. *difficile* strains. At 24 h of continuous measurement, the maximum OD_600_ of *C*. *difficile* ATCC 9689, ATCC 700057, and ATCC BAA-1870 treated with 1/4 MIC lipopeptide reached 51.57%, 51.54%, and 56.12% of the control. For the 1/2 MIC treatment, growth was reduced to 43.15%, 46.39%, and 46.12% of the control. For the 3/4 MIC treatment, growth was further reduced to 38.95%, 40.21%, and 41.84% of the control. The *C*. *difficile* ATCC9689 was significantly the most sensitive to the treatment (*P* < 0.01), and the inhibitory effect was stronger with an increased concentration of the C-1 lipopeptide (Figure 2).

Because *C*. *difficile* ATCC9689 was more sensitive to the C-1 lipopeptide, and it was also a *tcdA* and *tcdB* positive strain, we assessed the following antibacterial mechanism toward this strain.

### 3.3. Effect of C-1 Lipopeptide on *C. difficile* Morphology, Cell Wall Permeability

**Figure 3:**
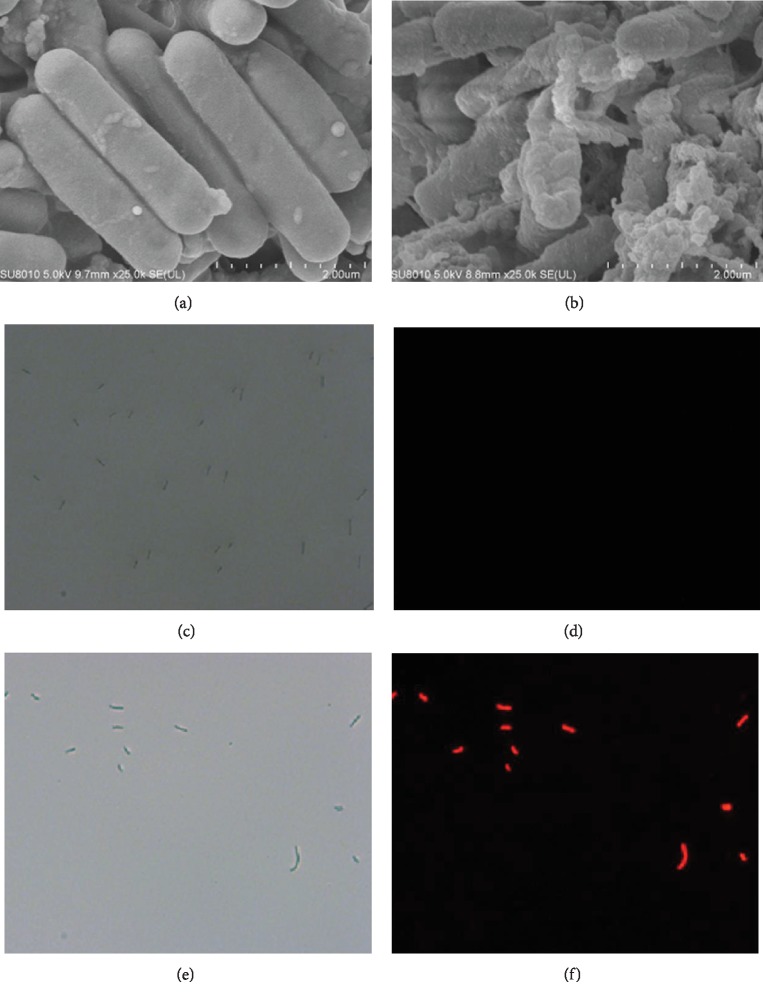
Morphological changes of *C*. *difficile* ATCC 9689 treated with 0.5 MIC of the C-1 lipopeptide. Observed with a scanning electron microscope (×25000; (a) untreated cells and (b) treated cells). Observed with a light microscope (×400; (c) untreated cells and (e) treated cells). Observed with a fluorescent microscope (PI staining, ×400; (d) untreated cells and (f) treated cells).

Scanning electron microscopy showed that the surface of *C*. *difficile* ATCC 9689 was damaged after treatment with the C-1 lipopeptide. Exudates surrounded the bacteria, and the cell wall and cell membrane were interrupted and indistinct, whereas untreated cells were smooth and uninterrupted (Figures 3(a) and 3(b)). In addition, with increasing concentrations of lipopeptide, the bacteria were surrounded by exudate that may have been cytoplasm extruded from the cells. Propidium iodide (PI) penetrates only a damaged cell membrane, after which it binds to double-stranded DNA and fluoresces red with illumination at 488 nm. We stained the C-1 lipopeptide-treated and C-1 lipopeptide-untreated *C*. *difficile* cells with PI and observed the cells with a fluorescence microscope. *C*. *difficile* ATCC 9689 cells treated with lipopeptide were stained with PI as shown by red fluorescence, which indicated a damaged cell membrane (Figures 3(d) and 3(f)), whereas the untreated cells did not show any fluorescence (Figures 3(c) and 3(e)).

The destroyed cell membrane increased the permeability of *C*. *difficile* ATCC 9689 after C-1 lipopeptide treatment. To verify the effect of lipopeptide on cell wall permeability, we measured alkaline phosphatase (AKPase) activity. A damaged cell wall and cell membrane increased cell permeability and caused an increase in extracellular AKPase. After lipopeptide treatment, extracellular AKPase activity increased continuously and was significantly higher than that of the untreated cells ([Supplementary-material supplementary-material-1]). With 36 h of incubation, the extracellular AKPase content of *C. difficile* ATCC 9689 treated with 1/4 MIC and 1/2 MIC of lipopeptide increased 4.7-fold and 7.7-fold.

The antibacterial activity of *Bacillus* spp. lipopeptide was observed with other pathogens, such as *S*. *aureus* [28], *Vibrio anguillarum* [18], and *E*. *clocae* [8]. However, our report is the first to document the effects of lipopeptide on toxin-producing *C*. *difficile*. The inhibitory mechanism of the C-1 lipopeptide against *C*. *difficile* could be explained by destroying the bacterial cell by forming ion-conducting channels in the cell membrane as described by Etchegaray et al. [29]. This mode of action drastically reduces the chance of the development of resistance by microbes, offering a promising alternative for the treatment of CDI.

### 3.4. Genome Sequencing of *B. amyloliquefaciens* C-1

**Figure 4:**
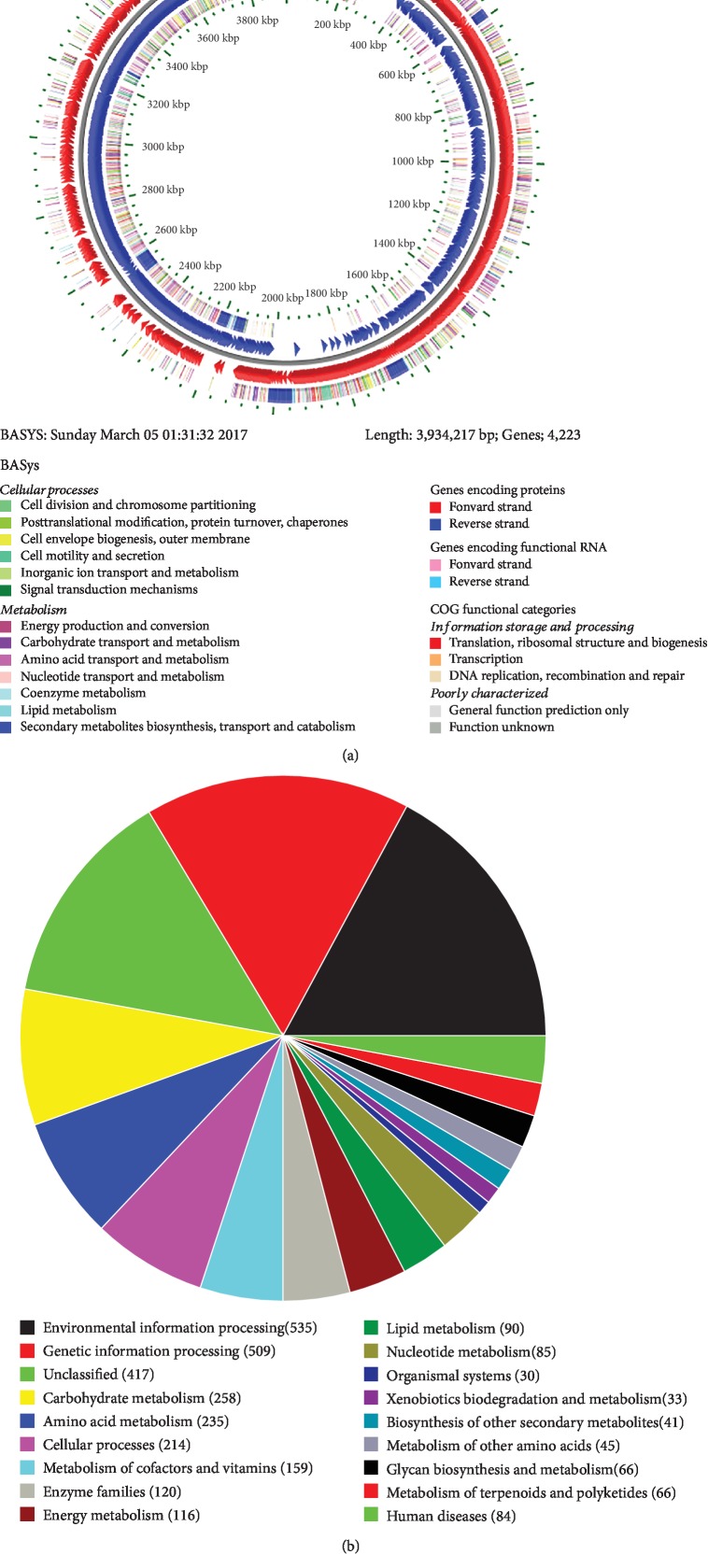
The genome map (a) of *B. amyloliquefaciens* C-1 and overview of the subsystem category coverage of the C-1 genome based on RAST serve (b). The red circle is the CDS of the forward strand, and the blue circle is the CDS of the reverse strand. The outer circle represents the categorization of predicted protein coding sequences in the C-1 genome in COG annotation, and the inner circle represents the genes encoding function RNA.

The circular chromosome of C-1 contains 3,934,216 bp, 46.5% GC content, 27 rRNA and 86 tRNA genes (Table 2, Figure 4(a)). Genome annotation at the RAST server showed that the C-1 genome encodes 4013 proteins, and the corresponding functional categorization by COG annotation is in Figure 4(b). The sequence data of the *B. amyloliquefaciens* C-1 genome were deposited into NCBI and can be accessed via accession number SRP127533.

Blast searches of the 16S rRNA gene sequence of C-1 showed that it was most similar to other *B*. *amyloliquefaciens* isolates. *B*. *amyloliquefaciens* isolates appear to group into two clades indicated by phylogenetic tree analysis ([Supplementary-material supplementary-material-1]). Although C-1 is in the same clade with known strains such as DSM7, ATCC19217, and ATCC 14580, it appears to be phylogenetically distant from most other isolates.

### 3.5. Secondary Metabolites from *B. amyloliquefaciens* Strains

As much as 8.5% of the *B. amyloliquefaciens* C-1 genome CDS was assigned to categories related to the secondary metabolites responsible for the control of pathogens (“Motility and Chemotaxis” [85 CDS], “Membrane Transport” [71 CDS], “Virulence, Disease and Defense” [70 CDS], “Secondary Metabolism” [6 CDS], and “Stress Responses” [108 CDS]). For the functional categories of genes, a possible role in bacteria inhibition may be important. In the carbohydrate transport and metabolism category, 437 genes (10.9% of total genes) were predicted in the C-1 genome. This finding suggests that C-1 possesses a broad battery of genes coding for enzymes required to release a variety of environmental carbon sources.

As a *Bacillus*. spp., *B*. *amyloliquefaciens* possesses an enormous potential to synthesize bioactive secondary metabolites, especially nonribosomal-synthesized peptides and polyketides. For the nonribosomal peptide synthetases (NRPSs) and polyketide synthases (PKS), we used antiSMASH to identify related giant gene clusters (Table 3, [Supplementary-material supplementary-material-1]). C-1 was found to harbor genes encoding six nonribosomal peptides (surfactins (srfABCD), iturins (ituABCD), fengycins (fenABCDE), bacillibactin (bmyABC), teichuronic, and bacilysin) and three polyketides (bacillaene (baeEDLMNJRS), difficidin (difABCDEFGHIJ), and macrolactin (mlnABCDEFGHI)). Compared with other sequenced *B*. *amyloliquefaciens* strains, fengycin, difficidin, bacillibactin, bacilysin, macrolactin, and bacillanen showed 100% identity, whereas surfactins had 82% identity ([Supplementary-material supplementary-material-1]). Surfactin can inhibit a wide range of microorganisms due to its ability to insert into the cell wall and create ion pores. Bacillomycin D, iturin, and fengycin have antifungal properties primarily based on their ability to disrupt the cell wall [30, 31]. Macrolactins, important 24-membered macrolactones produced by *Bacillus* spp., exhibit antimicrobial activities, where macrolactin A and E and succinyl macrolactin are the representative compounds. And it is assembled by a modular PKS system like macrolides, which could inhibit the H^+^-transporting ATPase of the bacterial cells [32]. Polyketide compounds inhibit a wide range of microorganisms by preventing protein synthesis [31]. The identity of surfactin among different strains varied from 82% to 96%, including subunit genes of SrfAA, SrfAB, SrfAC, and SrfAD ([Supplementary-material supplementary-material-1]). C-1 had all the subunit genes, SrfAA, SrfAB, SrfAC, and SrfAD, and the mutation of subunit genes in other strains may indicate loss of the ability to synthesize secondary metabolites [33].

PCR experiments detected C-1 surfactin, iturin, and fengycin genes, an intact Bac operon that included Bacilysin biosynthesis proteins BacA, BacB, BacC, BacD, and BacE (ORF3909-3913), and the oligopeptide permease operon (ORF1362-1366). Bac proteins, nonribosomally synthesized dipeptides active against a range of bacteria and some fungi, are involved in the biosynthesis of bacilysin. The proteolysis of this dipeptide releases the nonproteinogenic amino acid L-anticapsin, which functions as a competitive inhibitor of glucosamine synthase and can cause lysis of fungal cells [34]. Because there was 100% sequence identity of bacilysin, whereas there was no antifungal activity of the C-1 lipopeptide and exopolysaccharide, the regulation and expression of the encoded Bac operon, and modification of produced bacilysin, deserve more analysis.

Other antimicrobial gene clusters were predicted by antiSMASH, such as the lantibiotic amylolysin [35], the bacteriocin amylocyclicin [36], and the aminoglycoside antibiotic butirosin [37]. These antibacterials have not been detected by chemical analysis from C-1 supernatants, possibly because of the fermentation medium or condition that had been used [38]. Potential gene clusters may explain the broad activity of C-1 against pathogens.

We also detected in the C-1 genome other beneficial genes, such as phospholipase (ORF767) and siderophore production genes. There were seven gene clusters responsible for siderophore production and iron acquisition, including an ABC-type Fe^3+^-siderophore transport system (ORF1-2, ORF3417-3418, 4007-4008), an Fe-bacillibactin uptake system (ORF413-415), an iron compound ABC uptake transporter (ORF624-627), and a siderophore biosynthesis protein (ORF1221-1222, 3262-3267). These proteins enable bacteria to sequester iron complexes produced by other pathogens and antagonize certain pathogens [38, 39].

Moreover, we also detected genes for amphiphilic membrane-active biosurfactants and peptide antibiotics that have powerful antibacterial and mosquito larvicidal activity. The giant gene clusters add to the capacity of the C-1 bacterium to contribute to the antimicrobial activity against *C*. *difficile*. And we also checked the biosafety of the C-1 strain by using *Galleria mellonella* and intestinal epithelial cells, which all indicated the safety of *B. amyloliquefaciens* C-1. A future study of how these gene clusters are expressed and regulated will help explain the synthesis of antimicrobial lipopeptides and augment our knowledge for the control of CDI.

## 4. Conclusions

*B*. *amyloliquefaciens* C-1 fermentation supernatant contains a mixture of lipopeptides, namely, surfactin and fengycin, which had a strong inhibitory effect on *C. difficile* growth and viability. Systematic research of the antibacterial mechanism showed that the C-1 lipopeptide damages the integrity and permeability barrier of the cell wall and cell membrane, then leads to *C. difficile* cell death. The ~3.93 Mbp genome of C-1 reveals the genetic basis of its antimicrobial activity, and the antimicrobial compound-encoding gene clusters provide better understanding of the antibacterial mechanisms of this strain. Furthermore, the genome analysis will facilitate the production of effective probiotics that inhibit multidrug resistant pathogens in the host intestinal ecosystem, especially the phospholipase- and siderophore-producing clusters. Until now, the anti-*C*. *difficile* activities of the bacteriocins were known predominantly from *in vitro* studies; thus, the *in vivo* efficacies of the majority of these bacteriocins deserve further investigation.

## Figures and Tables

**Table 1:** The inhibition of the C-1 lipopeptide against *Clostridium difficile* in a plate test (the inhibition diameter showed in mm).

*Clostridium difficile*	Concentration (*μ*g/ml)			
	0	5	10	15
ATCC 9689	0.00	7.05 ± 0.71^a,b^	14.50 ± 0.71^a,b^	22.00 ± 1.41^a,b^
ATCC 700057	0.00	0.00	8.40 ± 0.71^a^	11.50 ± 0.71^a^
ATCC BAA-1870	0.00	0.00	8.50 ± 0.71^a^	10.50 ± 0.71^a^

^a^Significant difference of C-1 lipopeptide treatments vs. negative control (*P* < 0.01). ^b^Significant difference of lipopeptide treatments between *C. difficile* strains ATCC 9689 and ATCC 70057, ATCC 9689, and ATCC BAA-1870 (*P* < 0.01).

**Table 2:** Genome project information summary of *B. amyloliquefaciens* C-1.

Property/attributes	C-1	Property/attributes	C-1
Finishing quality	High-quality draft	Total predicted CDS	3805
Sequencing platform	PacBio Sequel	rRNA operons	27
Total bases (Mb)	757.4	tRNA	86
NCBI taxonomy ID	1386	tmRNA	1
BioProject ID	PRJNA427474	Noncoding RNA	81
Genome size (bp)	3934216	Miscellaneous RNA	81
GC content (%)	46.5		

**Table 3:** Identification of gene clusters potentially involved in the synthesis of secondary metabolites by *B. amyloliquefaciens* C-1.

Cluster^a^	Type^b^	From^c^	To^c^	Secondary metabolite^d^
1	Saccharide	165858	190731	Unknown
2	NRPS	556597	622004	Surfactin
3	Fatty acid	785309	810347	Unknown
4	NRPS	938299	967890	Iturins
5	Other KS	1158436	1199680	Butirosin
6	Fatty acid	1227747	1248724	Unknown
7	Terpene	1281720	1302460	Unknown
8	Fatty acid	1314519	1339344	Citrulline
9	Putative	1378928	1396630	Molybdenum cofactor
10	Lantipeptide	1406687	1451837	Unknown
11	Transatpks	1624433	1706630	Macrolactin
12	Transatpks-NRPS	1932737	2035409	Bacillaene
13	Transatpks-NRPS	2100037	2237835	Fengycin
14	Terpene	2263057	2284940	Unknown
15	T3PKS	2348257	2389357	Unknown
16	Transatpks	2504342	2604794	Difficidin
17	Bacteriocin-NRPS	3235204	3301995	Bacillibactin
18	Saccharide	3504667	3530078	Unknown
19	Saccharide	3624018	3678829	Teichuronic
20	Saccharide	3823278	3895655	Bacilysin

^a^Clusters identified using default settings of antiSMASH 5.0. ^b^Class of gene cluster according to antiSMASH 5.0. ^c^Location of gene clusters in the *B. amyloliquefaciens* C-1 genome. ^d^Secondary metabolites potentially produced based on the gene clusters.

## Data Availability

The data used to support the findings of this study are available from the corresponding author upon request.

## Supplementary Materials

Supplementary Materials Fig S1: the isolation, purification, and verification of the *B. amyloliquefaciens* C-1 lipopeptide. (A) The acid precipitated crude lipopeptide; (B) the UV-VIS spectrophotometer scanning analysis of crude lipopeptide; (C) the purified lipopeptide isolated by a TLC plate; (D) the PCR detection of lipopeptide synthesis-related genes fenB, srf, and ituD in the C-1 genome; phylogenetic tree of PCR fragments fenB (E), srf (F), and ituD (G). Fig S2: effect of the C-1 lipopeptide on AKPase in *C. difficile* ATCC 9689 (^∗^*P* < 0.05, ^∗∗^*P* < 0.01 indicated statistically significant differences of C-1 lipopeptide treatments vs. negative control). Fig S3: neighbor-joining phylogenetic tree based on 16S rRNA gene sequences of *B. amyloliquefaciens* strains. 16S rRNA gene sequences were from 16S ribosomal RNA gene partial sequence or directed from the genome annotation in NCBI with accession numbers of C-1 (JX028840), LL3 (CP002634.1), XH7 (CP002927.1), ATCC 13952 (CP009748.1), DSM7 (NC_014551), SRCM101267 (CP021505.1), Y2 (HE774679.1), UMAF6614 (NZ_CP006960), 19217 (CP009749.1), TA208 (CP002627.1), ATCC 14580 (CP000002.3), RD7-7 (CP016913.1), S499 (CP014700.1), LFB112 (NC_023073), CC178 (CP006845.1), L-H15 (CP010556.1), L-S60 (CP011278.1), B15 (KT923051.1), KHG19 (NZ_CP007242), ATCC 7050 (DQ297928.1), Y14 (NZ_CP017953), 168 (NC_000964), IT45 (NC_020272), ATCC 14581 (JQ579621.1), WS-8 (CP018200.1), LM2303 (MN640968.1), UMAF6639 (GCA_001593765), and DSM 319 (KM051080.1). Fig S4: phylogenetic tree of genome-sequenced *B. amyloliquefaciens* strains based on the amino acid sequences of surfactin synthetases SrfAA, SrfAB, SrfAC, and SrfAD, and the comparison of the gene loci strain C-1 (SRP127533), KHG19 (NZ_CP007242), UMAF6639 (GCA_001593765), UMAF6614 (NZ_CP006960), LFB112 (NC_023073), IT45 (NC_020272), DSM7 (NC_014551), Y14 (NZ_CP017953), and 168 (NC_000964). Table S1: comparison of gene clusters potentially involved in the synthesis of secondary metabolites in *B. amyloliquefaciens*-sequenced strains.
