# Supplementary material for: Mechanism of Antibacterial Activity of Bacillus amyloliquefaciens C-1 Lipopeptide toward Anaerobic Clostridium difficile
Source: Biomed Res Int. 2020 Mar 3;2020:3104613. doi: 10.1155/2020/3104613 (PMC7073505; doi:10.1155/2020/3104613)
Supplement: Supplementary Materials — Fig S1: the isolation, purification, and verification of the B. amyloliquefaciens C-1 lipopeptide. (A) The acid precipitated crude lipopeptide; (B) the UV-VIS spectrophotometer scanning analysis of crude lipopeptide; (C) the purified lipopeptide isolated by a TLC plate; (D) the PCR detection of lipopeptide synthesis-related genes fenB, srf, and ituD in the C-1 genome; phylogenetic tree of PCR fragments fenB (E), srf (F), and ituD (G). Fig S2: effect of the C-1 lipopeptide on AKPase in C. difficile ATCC 9689 (∗P < 0.05, ∗∗P < 0.01 indicated statistically significant differences of C-1 lipopeptide treatments vs. negative control). Fig S3: neighbor-joining phylogenetic tree based on 16S rRNA gene sequences of B. amyloliquefaciens strains. 16S rRNA gene sequences were from 16S ribosomal RNA gene partial sequence or directed from the genome annotation in NCBI with accession numbers of C-1 (JX028840), LL3 (CP002634.1), XH7 (CP002927.1), ATCC 13952 (CP009748.1), DSM7 (NC_014551), SRCM101267 (CP021505.1), Y2 (HE774679.1), UMAF6614 (NZ_CP006960), 19217 (CP009749.1), TA208 (CP002627.1), ATCC 14580 (CP000002.3), RD7-7 (CP016913.1), S499 (CP014700.1), LFB112 (NC_023073), CC178 (CP006845.1), L-H15 (CP010556.1), L-S60 (CP011278.1), B15 (KT923051.1), KHG19 (NZ_CP007242), ATCC 7050 (DQ297928.1), Y14 (NZ_CP017953), 168 (NC_000964), IT45 (NC_020272), ATCC 14581 (JQ579621.1), WS-8 (CP018200.1), LM2303 (MN640968.1), UMAF6639 (GCA_001593765), and DSM 319 (KM051080.1). Fig S4: phylogenetic tree of genome-sequenced B. amyloliquefaciens strains based on the amino acid sequences of surfactin synthetases SrfAA, SrfAB, SrfAC, and SrfAD, and the comparison of the gene loci strain C-1 (SRP127533), KHG19 (NZ_CP007242), UMAF6639 (GCA_001593765), UMAF6614 (NZ_CP006960), LFB112 (NC_023073), IT45 (NC_020272), DSM7 (NC_014551), Y14 (NZ_CP017953), and 168 (NC_000964). Table S1: comparison of gene clusters potentially involved in the synthesis of secondary metabolites in B. amyloliquefaciens-sequenced [file 3104613.f1.docx]

**Supplementary material**

**Fig. S1.** The isolation, purification and verification of *B.amyloliquefaciens* C-1 lipopeptide. A, the acid precipitated crude lipopeptide; B, the UV-VIS spectrophotometer scanning analysis of crude lipopeptide; C, the purified lipopeptide isolated by TLC plate; D, the PCR detection of lipopeptide synthesis related genes *fenB*, *srf*, *ituD* in C-1 genome; Phylogenetic tree of PCR fragments *fenB*(E), *srf*(F), *ituD*(G).

**Fig. S2.** Effect of C-1 lipopeptide on AKPase in *C.difficile* ATCC 9689 (**P*<0.05, ** *P*<0.01 indicated statically significant differences of C-1 lipopeptide treatments vs. negative control).

**Fig. S3.** Neighbor-joining phylogenetic tree based on 16S rRNA gene sequences of *B. amyloliquefaciens* strains. 16S rRNA gene sequences were from 16S ribosomal RNA gene partial sequence or directed from the genome annotation in NCBI with accession number of C-1 (JX028840), LL3 (CP002634.1), XH7 (CP002927.1), ATCC 13952 (CP009748.1), DSM7 (NC_014551), SRCM101267 (CP021505.1), Y2 (HE774679.1), UMAF6614 (NZ_CP006960), 19217 (CP009749.1), TA208 (CP002627.1), ATCC 14580 (CP000002.3), RD7-7 (CP016913.1), S499 (CP014700.1), LFB112 (NC_023073), CC178 (CP006845.1), L-H15 (CP010556.1), L-S60 (CP011278.1), B15 (KT923051.1), KHG19 (NZ_CP007242), ATCC 7050 (DQ297928.1), Y14 (NZ_CP017953), 168 (NC_000964), IT45 (NC_020272), ATCC 14581 (JQ579621.1), WS-8 (CP018200.1), LM2303 (MN640968.1), UMAF6639 (GCA_001593765), DSM 319 (KM051080.1)

**Fig. S4.** Phylogenetic tree of genome-sequenced *B. amyloliquefaciens* strains based on the amino acids sequences of Surfactin synthetase SrfAA, SrfAB, SrfAC and SrfAD, and the comparison of the genes locus. Strain C-1 (SRP127533), KHG19 (NZ_CP007242), UMAF6639 (GCA_001593765), UMAF6614 (NZ_CP006960), LFB112 (NC_023073), IT45 (NC_020272), DSM7 (NC_014551), Y14 (NZ_CP017953), 168 (NC_000964).

**Table S1** Comparison of gene clusters potentially involved in the synthesis of secondary metabolites in *B. amyloliquefaciencs* sequenced strains.

**Figure S1**.


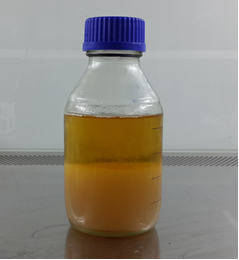
A


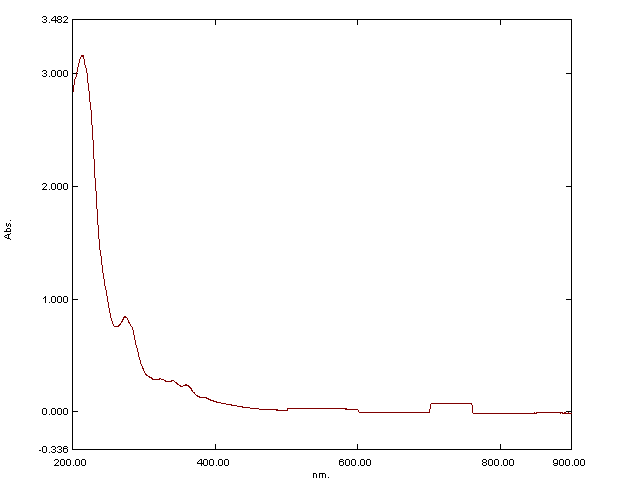
 B


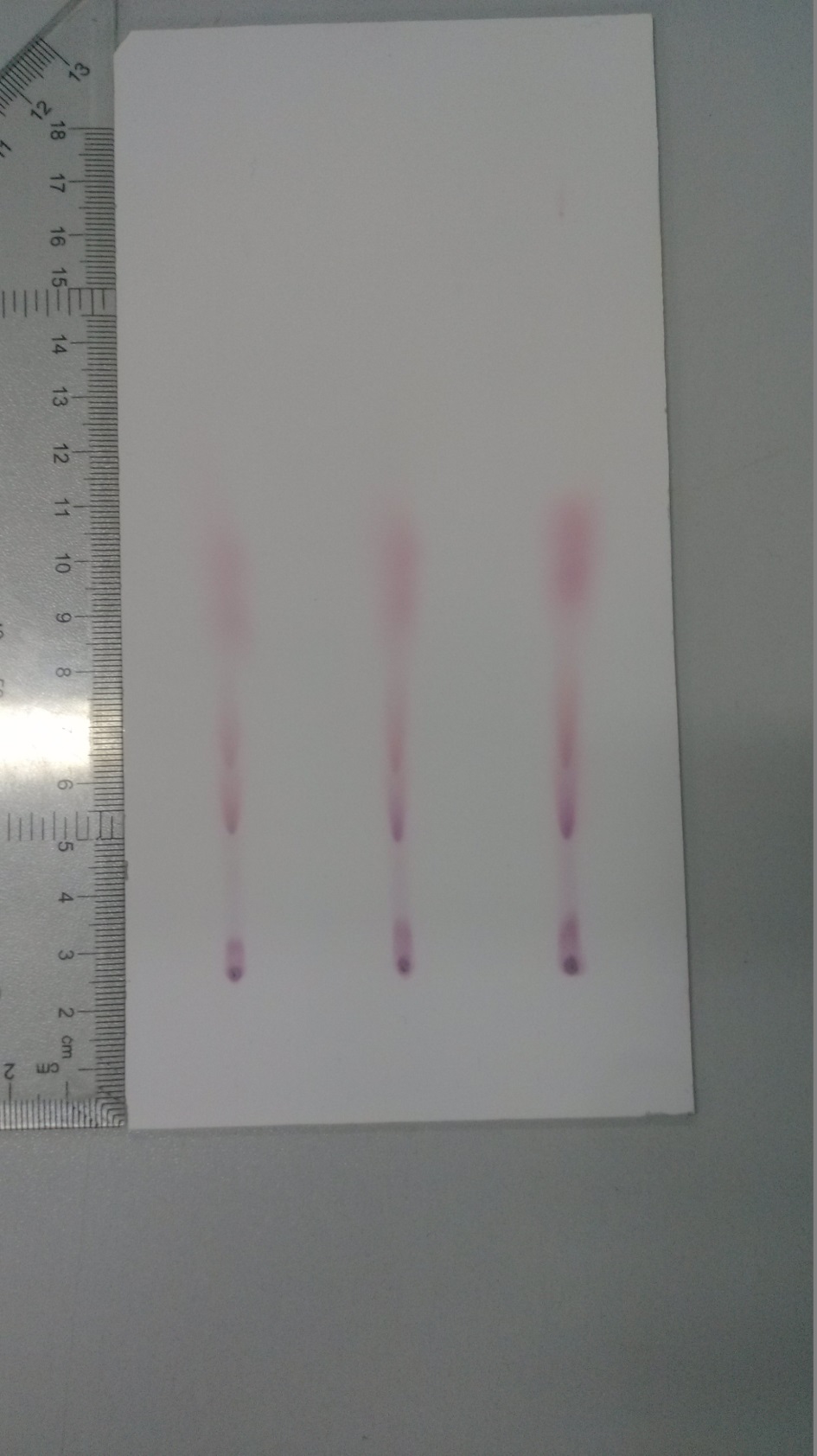
C


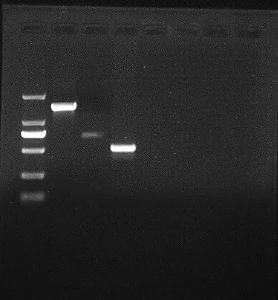
 D

M *fenB srf ituD* -

2000bp

1000bp

750bp

500bp

250bp

100bp

EF

G

**Figure S2**.


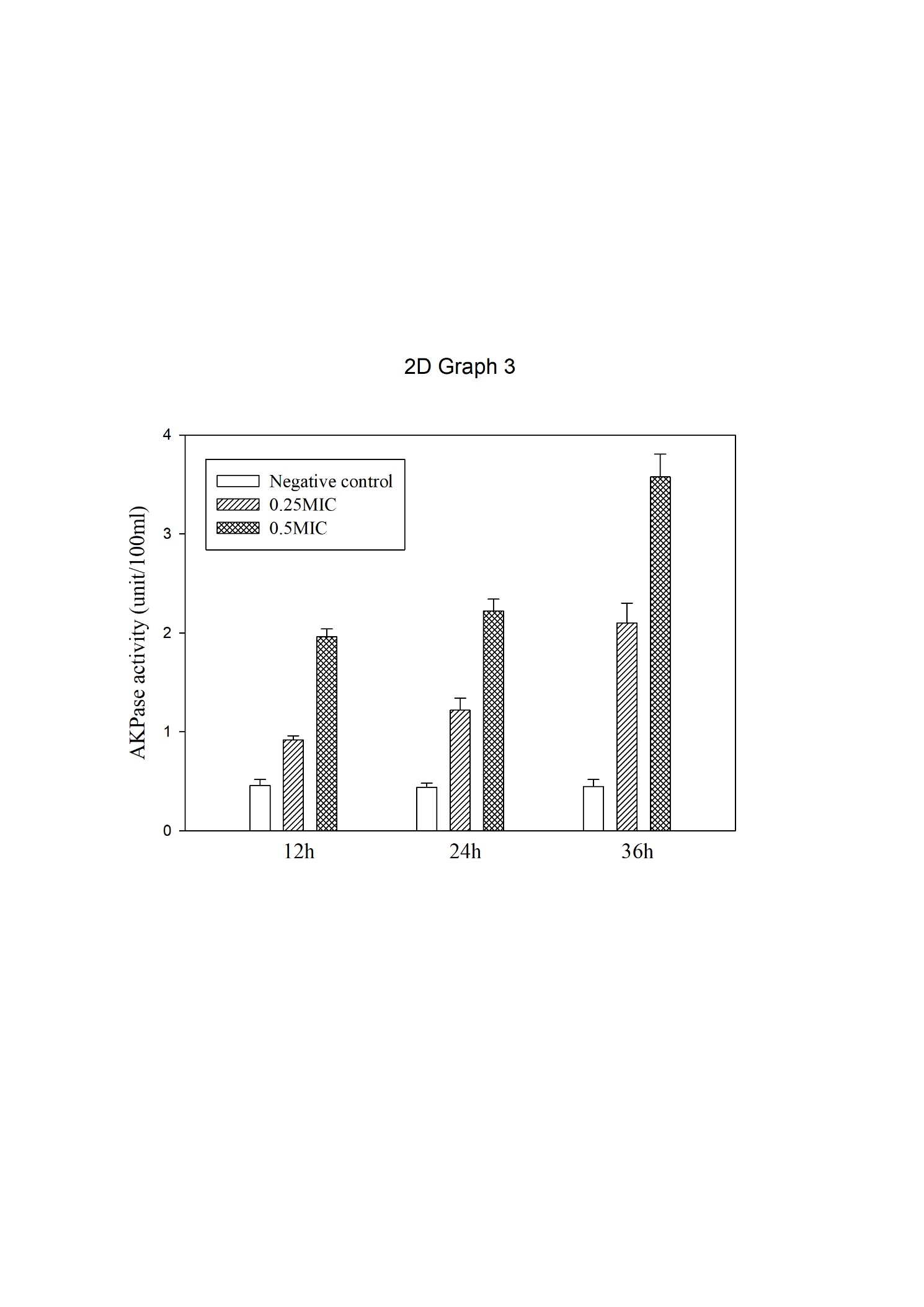
A

**

**

**

**

**

*

**Figure S3**.





**Figure S4**.


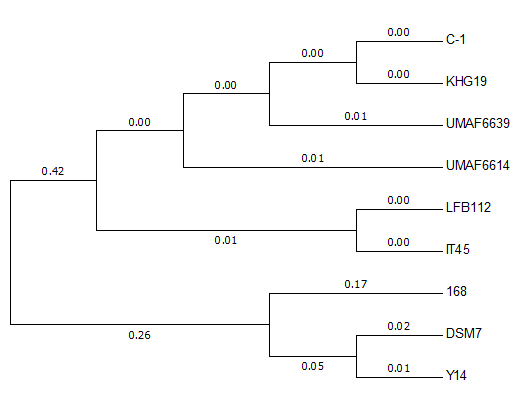


SrfAA

SrfAB

SrfAC

SrfAD

SrfAA

SrfAB

SrfAD

SrfAB

SrfAC

SrfAD

SrfAA

SrfAB

SrfAC

SrfAD

SrfAC

SrfAD

SrfAA

SrfAB

SrfAC

SrfAD

SrfAA

SrfAB

SrfAC

SrfAD

SrfAA

SrfAB

SrfAC

SrfAD

SrfAA

SrfAC

SrfAD

**Table S1** Comparison of gene clusters potentially involved in the synthesis of secondary metabolites in *B.amyloliquesfaciencs* sequenced strains

| Features | *B.amyloliquefaciens* | | | | | | | | *B.subtilis* subsp. subtilis str.168 |
| --- | --- | --- | --- | --- | --- | --- | --- | --- | --- |
|  | C-1 | DSM 7 | UMAF6639 | UMAF6614 | LFB112 | KHG19 | Y14 | IT45 |  |
| General features | | | | |  |  |  |  |  |
| GC% | 46.5% | 46.1% | 46.3% | 46.5% | 46.7% | 46.6% | 46.4% | 46.6% | 43.5% |
| Genome size (bp) | 3934216 | 3980199 | 4034636 | 4005145 | 3942754 | 3953361 | 3957164 | 3928857 | 4215606 |
| proteins | 3805 | 3870 | 3741 | 3754 | 3684 | 3698 | 3741 | 3726 | 4237 |
| rRNA | 27 | 30 | 27 | 27 | 31 | 28 | 27 | 30 | 30 |
| rRNA | 86 | 94 | 82 | 82 | 94 | 88 | 87 | 95 | 86 |
| Gene | 3805 | 4120 | 3955 | 3947 | 3900 | 3899 | 3964 | 3936 | 4536 |
| Non-ribosomal synthesis of lipopeptides and polyketides | | | | |  |  |  |  |  |
| Fengycin | 100% | 93% | 100% | 100% | 100% | 100% | 100% | 100% | 100% |
| Difficidin | 100% | — | 100% | 100% | 100% | 100% | 100% | 100% | — |
| Bacillibactin | 100% | 100% | 100% | 100% | 100% | 100% | 100% | 100% | 100% |
| Bacilysin | 100% | 85% | 100% | 100% | 100% | 100% | 100% | 100% | 100% |
| Rhizocticin | — | — | 22% | — | 22% | — | — | — | — |
| Surfactin | 82% | 82% | 91% | 86% | 82% | 95% | 82% | 82% | 86% |
| Kijanimicin | — | — | 4% | — | — | — | — | — | 100% |
| Butirosin | 7% | 7% | 7% | 7% | 7% | 7% | 7% | 7% | — |
| Macrolactin | 100% | — | 100% | 100% | 100% | 100% | 100% | — | — |
| Bacillaene | 100% | 100% | 92% | 100% | 100% | 100% | 100% | — | 100% |
| subtilosin | — | — | — | — | — | — | — | — | 100% |
| Mersacidin | — | — | — | — | — | 100% | — | — | — |
| Plantathiazolicin |  |  |  |  |  |  | 100% | — | — |
